# Supplementary material for: Comparison of Ferroptosis-Inhibitory Mechanisms between Ferrostatin-1 and Dietary Stilbenes (Piceatannol and Astringin)
Source: Molecules. 2021 Feb 19;26(4):1092. doi: 10.3390/molecules26041092 (PMC7922211; doi:10.3390/molecules26041092)
Supplement: Supplementary file 1 [file molecules-26-01092-s001.zip › Suppls/Suppl. 2 The partial calculation results of neutral molecules.pdf]

Supplementary Material-2 The partial calculation results of neutral molecules

Comparison of Ferroptosis-inhibitory Mechanisms between Ferrostatin-1 and Dietary Stilbenes (Piceatannol and Astringin)

Ban Chen [1], Xican Li [1,*], Xiaojian Ouyang [1], Jie Liu [2,3], Yangping Liu [4] Dongfeng Chen[2,*]

[1] School of Chinese Herbal Medicine; Guangzhou University of Chinese Medicine, Guangzhou 510006, China; imchenban@foxmail.com (B.C.); oyxiaojian55@163.com (X.O.)

[2] School of Basic Medical Science, Guangzhou University of Chinese Medicine, Guangzhou 510006, China; 15014173165@163.com (J.L.)

[3.] The Research Center of Basic Integrative Medicine, Guangzhou University of Chinese Medicine, Guangzhou 510006, China.

[4.] The Fourth Clinical Medical College, Guangzhou University of Chinese Medicine, Waihuan East Road No. 232, Guangzhou Higher Education Mega Center, Guangzhou 510006, China; 20182119168@stu.gzucm.edu.cn (Y.L.)

* Correspondence: lixc@gzucm.edu.cn (X.L.); lixican@126.com (X.L.); chen888@gzucm.edu.cn (D.C.). Tel: +86-020-39358076; Fax: +86-020-38892690

Table S2.1 The partial calculation results of Ferrostatin-1

Ferrostatin-1 calculated at M06-2X-D3/6-311+G** level in methanol SMD solvation model					
Cartesian coordinates of optimized molecule				Thermal properties /(Hartree)	
Symbol	X	Y	Z	Zero-point correction	0.358188
C	3.785627	-1.237323	1.669815	Thermal correction to Energy	0.376542
C	4.936856	-0.234628	1.575344	Thermal correction to Enthalpy	0.377486
C	4.171802	0.411268	-0.736583	Thermal correction to Gibbs Free Energy	0.310586
C	3.031282	-0.604455	-0.643158	Sum of electronic and zero-point Energies	-844.367961
C	2.594199	-0.806794	0.808483	Sum of electronic and thermal Energies	-844.349607
C	-0.391702	-1.044722	-1.271547	Sum of electronic and thermal Enthalpies	-844.348663
C	0.604198	-0.085292	-1.081477	Sum of electronic and thermal Free Energies	-844.415563
N	1.910929	-0.277445	-1.582571	Single point energy at def2-TZVPD basis set	-844.8137027
C	-1.691085	-0.848413	-0.822135		
C	-2.003574	0.335094	-0.153642		
C	-1.02705	1.316065	0.025782		
C	0.270197	1.132308	-0.445253		
C	-3.365652	0.602529	0.370131		
O	-4.232165	-0.373092	0.104676		
C	-5.583443	-0.194543	0.585071		
C	-6.366582	-1.417315	0.175224		
N	1.243773	2.113244	-0.275534		
O	-3.676628	1.605347	0.982019		
C	5.361502	-0.029184	0.120302		
H	3.464464	-1.352597	2.708825		
H	4.136705	-2.220544	1.333035		
H	4.612541	0.726269	1.993948		
H	5.785324	-0.575913	2.175477		
H	4.469721	0.523807	-1.783289		
H	3.823604	1.388022	-0.384424		
H	3.432154	-1.56436	-0.991002		
H	2.18307	0.129511	1.201763		
H	1.80271	-1.560622	0.857465		
H	-0.127037	-1.968636	-1.776122		
H	1.878667	-0.997085	-2.29499		
H	-2.442002	-1.612582	-0.975025		
H	-1.284829	2.248585	0.516768		
H	-5.550579	-0.074981	1.669576		
H	-5.988786	0.717162	0.142378		
H	-7.397076	-1.318486	0.522305		
H	-6.376635	-1.525405	-0.911227		
H	-5.937778	-2.318633	0.617871		
H	0.864813	3.043913	-0.148174		
H	1.952061	2.094524	-1.001507		

H	6.162719	0.712274	0.055721		
H	5.764159	-0.970444	-0.274323		
Ferrostatin-1 calculated at M06-2X-D3/6-311+G** level in aqueous SMD solvation model					
Cartesian coordinates of optimized molecule				Thermal properties /(Hartree)	
Symbol	X	Y	Z	Zero-point correction	0.358268
C	3.783834	-1.281385	1.639738	Thermal correction to Energy	0.376623
C	4.916914	-0.255817	1.584271	Thermal correction to Enthalpy	0.377568
C	4.158778	0.447719	-0.712533	Thermal correction to Gibbs Free Energy	0.310677
C	3.036447	-0.590073	-0.658742	Sum of electronic and zero-point Energies	-844.358591
C	2.592474	-0.845487	0.78185	Sum of electronic and thermal Energies	-844.340236
C	-0.386629	-1.033411	-1.280897	Sum of electronic and thermal Enthalpies	-844.339291
C	0.609869	-0.075245	-1.08519	Sum of electronic and thermal Free Energies	-844.406182
N	1.912581	-0.269752	-1.598899	Single point energy at def2-TZVPD basis set	-844.8044063
C	-1.685366	-0.842787	-0.82882		
C	-1.99864	0.335326	-0.151104		
C	-1.02122	1.313564	0.035698		
C	0.276216	1.134727	-0.436189		
C	-3.360015	0.599278	0.374488		
O	-4.228292	-0.374992	0.102925		
C	-5.579341	-0.195623	0.585313		
C	-6.367981	-1.411988	0.167812		
N	1.244406	2.119614	-0.251327		
O	-3.671026	1.59899	0.993488		
C	5.348663	-0.002464	0.139201		
H	3.457228	-1.434575	2.671639		
H	4.155197	-2.246307	1.274252		
H	4.570854	0.686772	2.025637		
H	5.76627	-0.598159	2.18166		
H	4.464146	0.597371	-1.751789		
H	3.79665	1.407387	-0.330817		
H	3.460891	-1.529252	-1.031534		
H	2.161035	0.070554	1.20032		
H	1.813919	-1.613677	0.799344		
H	-0.12187	-1.951995	-1.79439		
H	1.866833	-1.016132	-2.282432		
H	-2.434867	-1.6065	-0.987798		
H	-1.276884	2.242309	0.534299		
H	-5.545017	-0.084182	1.670156		
H	-5.981236	0.720226	0.149308		
H	-7.396991	-1.310133	0.516885		
H	-6.379939	-1.512194	-0.918891		
H	-5.943181	-2.317808	0.603876		
H	0.853771	3.046253	-0.131565		

H	1.96013	2.10728	-0.969247		
H	6.14004	0.750604	0.101619		
H	5.764641	-0.926882	-0.279627		

Table S2.2 The partial calculation results of piceatannol

Piceatannol calculated at M06-2X-D3/6-311+G** level in methanol SMD solvation model					
Cartesian coordinates of optimized molecule				Thermal properties /(Hartree)	
Symbol	X	Y	Z	Zero-point correction	0.232282
C	-0.381757	0.298187	-0.011406	Thermal correction to Energy	0.248535
C	0.627627	-0.583002	-0.040316	Thermal correction to Enthalpy	0.249479
C	-1.815117	-0.025904	-0.025349	Thermal correction to Gibbs Free Energy	0.187300
C	2.061153	-0.252488	-0.026584	Sum of electronic and zero-point Energies	-841.309835
C	-2.732209	1.026636	0.099827	Sum of electronic and thermal Energies	-841.293582
C	-2.310634	-1.328993	-0.15998	Sum of electronic and thermal Enthalpies	-841.292638
C	2.972718	-1.306907	0.064253	Sum of electronic and thermal Free Energies	-841.354817
C	2.537221	1.065093	-0.100476	Single point energy at def2-TZVPD basis set	-841.6364985
C	-4.097085	0.790216	0.096687		
C	-3.678673	-1.56849	-0.161977		
C	4.341484	-1.04546	0.089607		
C	3.904652	1.300794	-0.073725		
O	-4.959815	1.839642	0.220795		
C	-4.576788	-0.516134	-0.03378		
O	5.178263	-2.118479	0.185051		
C	4.823078	0.254997	0.021049		
O	4.421194	2.561794	-0.140699		
O	-5.933603	-0.659799	-0.027345		
H	-0.161652	1.361875	0.035245		
H	0.411727	-1.647855	-0.064394		
H	-2.384742	2.049676	0.202644		
H	-1.635642	-2.169201	-0.268407		
H	2.627993	-2.33336	0.120159		
H	1.861399	1.908756	-0.184225		
H	-4.064384	-2.577492	-0.266842		
H	-5.870245	1.513532	0.197211		
H	6.098877	-1.827092	0.202151		
H	5.88783	0.461547	0.038668		
H	3.707532	3.209872	-0.200587		
H	-6.181013	-1.586787	-0.141013		
Piceatannol calculated at M06-2X-D3/6-311+G** level in aqueous SMD solvation model					
Cartesian coordinates of optimized molecule				Thermal properties /(Hartree)	
Symbol	X	Y	Z	Zero-point correction	0.232129
C	-0.380876	0.296798	-0.010642	Thermal correction to Energy	0.248362
C	0.627485	-0.585484	-0.039011	Thermal correction to Enthalpy	0.249307
C	-1.814084	-0.026984	-0.023253	Thermal correction to Gibbs Free Energy	0.187262
C	2.060592	-0.254187	-0.026168	Sum of electronic and zero-point Energies	-841.304918
C	-2.730256	1.026992	0.094174	Sum of electronic and thermal Energies	-841.288685
C	-2.309498	-1.331051	-0.149015	Sum of electronic and thermal Enthalpies	-841.287741

C	2.973325	-1.308261	0.059437	Sum of electronic and thermal Free Energies	-841.349785
C	2.535023	1.063954	-0.095387	Single point energy at def2-TZVPD basis set	-841.6312705
C	-4.094789	0.790151	0.091169		
C	-3.677415	-1.570081	-0.15127		
C	4.340876	-1.044126	0.083907		
C	3.901967	1.300297	-0.06972		
O	-4.962161	1.844566	0.208286		
C	-4.574603	-0.516027	-0.031606		
O	5.185451	-2.119253	0.175194		
C	4.822274	0.256435	0.019269		
O	4.415551	2.569345	-0.13245		
O	-5.936024	-0.662816	-0.02673		
H	-0.160511	1.360356	0.033558		
H	0.41162	-1.650185	-0.062492		
H	-2.381058	2.04972	0.190087		
H	-1.634037	-2.171528	-0.249572		
H	2.628478	-2.334484	0.111925		
H	1.858601	1.907467	-0.173221		
H	-4.064994	-2.578687	-0.249255		
H	-5.869545	1.510932	0.184953		
H	6.102119	-1.816302	0.193822		
H	5.886968	0.461549	0.036552		
H	3.693282	3.208405	-0.183922		
H	-6.173576	-1.592524	-0.138142		

Table S2.3 The partial calculation results of astringin

Astringin calculated at M06-2X-D3/6-311+G** level in methanol SMD solvation model					
Cartesian coordinates of optimized molecule				Thermal properties /(Hartree)	
Symbol	X	Y	Z	Zero-point correction	0.408893
C	-3.854124	0.327293	-0.880816	Thermal correction to Energy	0.435952
O	-2.535514	-0.149322	-1.083274	Thermal correction to Enthalpy	0.436897
O	-4.075947	0.741908	0.441277	Thermal correction to Gibbs Free Energy	0.351563
C	-4.801207	-0.788605	-1.326123	Sum of electronic and zero-point Energies	-1451.878359
C	-1.480982	0.68748	-0.835879	Sum of electronic and thermal Energies	-1451.851299
C	-4.127306	-0.26453	1.467166	Sum of electronic and thermal Enthalpies	-1451.850355
C	-4.886142	-1.931439	-0.301145	Sum of electronic and thermal Free Energies	-1451.935689
O	-6.083557	-0.239646	-1.57181	Single point energy at def2-TZVPD basis set	-1452.4415739
C	-0.226275	0.081873	-0.837587		
C	-1.619964	2.050287	-0.587382		
C	-5.107427	-1.380605	1.107001		
C	-2.738354	-0.736831	1.886742		
O	-3.704367	-2.708902	-0.258355		
C	0.920489	0.839741	-0.588804		
C	-0.462539	2.79761	-0.349914		
O	-6.442818	-0.880155	1.10887		
O	-1.977984	0.349687	2.403884		
C	2.218644	0.14795	-0.589802		
C	0.795468	2.213975	-0.347407		
O	-0.528918	4.138222	-0.110189		
C	3.381723	0.670111	-0.177111		
C	4.680668	-0.01733	-0.179802		
C	5.745862	0.57036	0.515844		
C	4.905335	-1.227911	-0.846576		
C	6.989879	-0.037229	0.562693		
C	6.15252	-1.83801	-0.802735		
C	7.196671	-1.251712	-0.098315		
O	8.001267	0.561597	1.254755		
O	8.45104	-1.780393	-0.004927		
H	-4.030914	1.212605	-1.496062		
H	-4.433762	-1.182044	-2.276475		
H	-4.543294	0.270458	2.326243		
H	-5.749983	-2.550499	-0.565297		
H	-6.516165	-0.090029	-0.717417		
H	-0.157613	-0.983295	-1.028627		
H	-2.58476	2.541361	-0.567248		
H	-5.002249	-2.196048	1.829242		
H	-2.203538	-1.2283	1.073355		
H	-2.855061	-1.450986	2.704502		

H	-3.548929	-3.094627	-1.129028		
H	-6.637641	-0.490501	1.969899		
H	-1.81666	0.980945	1.69017		
H	2.18501	-0.879843	-0.941633		
H	1.658069	2.843122	-0.164855		
H	-1.445823	4.44072	-0.12799		
H	3.403658	1.681862	0.220601		
H	5.609758	1.512671	1.036884		
H	4.113179	-1.699074	-1.415804		
H	6.330687	-2.774293	-1.322023		
H	8.798004	0.015993	1.200926		
H	8.504774	-2.619047	-0.481483		
Astringin calculated at M06-2X-D3/6-311+G** level in aqueous SMD solvation model					
Cartesian coordinates of optimized molecule				Thermal properties /(Hartree)	
Symbol	X	Y	Z	Zero-point correction	0.408866
C	-3.863986	0.334769	-0.88236	Thermal correctio n to Energy	0.436084
O	-2.54708	-0.137667	-1.108184	Thermal correction to Enthalpy	0.437028
O	-4.067761	0.742347	0.446787	Thermal correction to Gibbs Free Energy	0.351168
C	-4.81406	-0.782134	-1.317611	Sum of electronic and zero-point Energies	-1451.873802
C	-1.49012	0.695675	-0.848642	Sum of electronic and thermal Energies	-1451.846584
C	-4.09707	-0.270494	1.467243	Sum of electronic and thermal Enthalpies	-1451.845640
C	-4.878324	-1.931238	-0.298134	Sum of electronic and thermal Free Energies	-1451.931500
O	-6.103863	-0.236836	-1.536312	Single point energy at def2-TZVPD basis set	-1452.4367737
C	-0.237565	0.086071	-0.841347		
C	-1.627555	2.057744	-0.596997		
C	-5.07543	-1.391105	1.117633		
C	-2.70001	-0.740274	1.861727		
O	-3.696961	-2.712027	-0.286326		
C	0.909086	0.839578	-0.579714		
C	-0.470172	2.798944	-0.343504		
O	-6.415659	-0.901894	1.149479		
O	-1.929785	0.34687	2.365088		
C	2.205414	0.145066	-0.577739		
C	0.785903	2.212715	-0.330803		
O	-0.537864	4.144541	-0.097368		
C	3.369179	0.666979	-0.166839		
C	4.667379	-0.021209	-0.171805		
C	5.737693	0.574932	0.508303		
C	4.886545	-1.239119	-0.827197		
C	6.981984	-0.031739	0.549869		
C	6.134539	-1.847457	-0.788705		
C	7.184106	-1.251904	-0.100278		
O	8.004549	0.576614	1.229361		

O	8.443727	-1.781959	-0.014328		
H	-4.05168	1.223359	-1.488651		
H	-4.461641	-1.170025	-2.275554		
H	-4.502027	0.256103	2.336337		
H	-5.74663	-2.547902	-0.551169		
H	-6.518256	-0.102221	-0.670671		
H	-0.169051	-0.978185	-1.035754		
H	-2.590269	2.552742	-0.587883		
H	-4.949358	-2.20932	1.832795		
H	-2.179688	-1.230335	1.038392		
H	-2.802837	-1.455277	2.679946		
H	-3.547513	-3.060111	-1.173584		
H	-6.582952	-0.495181	2.008158		
H	-1.777984	0.971219	1.643219		
H	2.170157	-0.882457	-0.929563		
H	1.648933	2.837083	-0.136086		
H	-1.456209	4.440983	-0.128491		

Table S3.4 The partial calculation results of cytosine, adenine, and guanine

Cytosine calculated at M06-2X-D3/6-311+G** level in aqueous SMD solvation model					
Cartesian coordinates of optimized molecule				Thermal properties /(Hartree)	
Symbol	X	Y	Z	Zero-point correction	0.098972
N	-1.277274	0.885239	-0.000408	Thermal correction to Energy	0.105594
C	-1.172252	-0.497976	0.000001	Thermal correction to Enthalpy	0.106539
O	-2.214319	-1.171305	-0.000007	Thermal correction to Gibbs Free Energy	0.068305
N	0.062698	-1.050556	0.00051	Sum of electronic and zero-point Energies	-394.829026
C	1.133801	-0.258962	0.000098	Sum of electronic and thermal Energies	-394.822404
N	2.340002	-0.838161	-0.000073	Sum of electronic and thermal Enthalpies	-394.821460
C	1.04922	1.171107	0.000219	Sum of electronic and thermal Free Energies	-394.859694
C	-0.192625	1.700688	0.000033	Single point energy at def2-TZVPD basis set	-394.9725806
H	-2.211417	1.277331	-0.000567		
H	2.41607	-1.844755	-0.001446		
H	3.179651	-0.279061	-0.001079		
H	1.935566	1.788168	0.000519		
H	-0.392172	2.763958	0.000324		
Adenine calculated at M06-2X-D3/6-311+G** level in aqueous SMD solvation model					
Cartesian coordinates of optimized molecule				Thermal properties /(Hartree)	
Symbol	X	Y	Z	Zero-point correction	0.112865
N	1.954137	-0.472269	0.000004	Thermal correction to Energy	0.120137
C	1.339431	-1.667449	0.001152	Thermal correction to Enthalpy	0.121081
N	0.044234	-1.930295	0.002199	Thermal correction to Gibbs Free Energy	0.081104
C	-0.718262	-0.818152	0.001631	Sum of electronic and zero-point Energies	-467.185678
C	-0.191016	0.469621	-0.000782	Sum of electronic and thermal Energies	-467.178405
C	1.202874	0.634703	-0.002459	Sum of electronic and thermal Enthalpies	-467.177461
N	1.80314	1.843638	-0.043964	Sum of electronic and thermal Free Energies	-467.217438
N	-2.092006	-0.769056	0.002452	Single point energy at def2-TZVPD basis set	-467.3512634
C	-2.372917	0.516885	0.000129		
N	-1.278513	1.312944	-0.001881		
H	2.004962	-2.523583	0.002758		
H	1.26578	2.673972	0.159218		
H	2.79699	1.881003	0.133925		
H	-3.367929	0.93493	-0.000428		
H	-1.277415	2.325288	-0.005171		
Guanine calculated at M06-2X-D3/6-311+G** level in aqueous SMD solvation model					
Cartesian coordinates of optimized molecule				Thermal properties /(Hartree)	
Symbol	X	Y	Z	Zero-point correction	0.117547
N	1.739165	-1.486974	0.001485	Thermal correction to Energy	0.125758
C	2.702896	-0.51109	0.00275	Thermal correction to Enthalpy	0.126702
N	2.20507	0.6958	0.001038	Thermal correction to Gibbs Free Energy	0.084548
C	0.532421	-0.855383	-0.001379	Sum of electronic and zero-point Energies	-542.435089
N	-0.684759	-1.442403	0.001174	Sum of electronic and thermal Energies	-542.426878

C	-1.668495	-0.572417	-0.002349	Sum of electronic and thermal Enthalpies	-542.425934
N	-2.952309	-0.992007	-0.053814	Sum of electronic and thermal Free Energies	-542.468087
N	-1.472226	0.785044	-0.001601	Single point energy at def2-TZVPD basis set	-542.613847
C	-0.233542	1.433536	-0.000249		
O	-0.179404	2.663781	0.00223		
C	0.837584	0.496952	-0.001709		
H	1.897622	-2.487315	0.002382		
H	3.752379	-0.760314	0.005048		
H	-3.110241	-1.96918	0.148595		
H	-3.686952	-0.351589	0.214332		
H	-2.287351	1.392335	-0.008555		
