# Supplementary material for: Comparison of Ferroptosis-Inhibitory Mechanisms between Ferrostatin-1 and Dietary Stilbenes (Piceatannol and Astringin)
Source: Molecules. 2021 Feb 19;26(4):1092. doi: 10.3390/molecules26041092 (PMC7922211; doi:10.3390/molecules26041092)
Supplement: Supplementary file 1 [file molecules-26-01092-s001.zip › Suppls/Suppl. 1 Dose response curves.pdf]

Supplementary Material-1 Dose response curves

Comparison of Ferroptosis-inhibitory Mechanisms between Ferrostatin-1 and Dietary Stilbenes (Piceatannol and Astringin)

Ban Chen [1], Xican Li [1,*], Xiaojian Ouyang [1], Jie Liu [2,3], Yangping Liu [4] Dongfeng Chen[2,*]

[1] School of Chinese Herbal Medicine; Guangzhou University of Chinese Medicine, Guangzhou 510006, China; imchenban@foxmail.com (B.C.); oyxiaojian55@163.com (X.O.)

[2] School of Basic Medical Science, Guangzhou University of Chinese Medicine, Guangzhou 510006, China; 15014173165@163.com (J.L.)

[3.] The Research Center of Basic Integrative Medicine, Guangzhou University of Chinese Medicine, Guangzhou 510006, China.

[4.] The Fourth Clinical Medical College, Guangzhou University of Chinese Medicine, Waihuan East Road No. 232, Guangzhou Higher Education Mega Center, Guangzhou 510006, China; 20182119168@stu.gzucm.edu.cn (Y.L.)

* Correspondence: lixc@gzucm.edu.cn (X.L.); lixican@126.com (X.L.); chen888@gzucm.edu.cn (D.C.). Tel: +86-020-39358076; Fax: +86-020-38892690

Note: This Supporting Information provides the original data of Table 1 in the main text. All data with underline are mentioned in **Table 1**.

1. ${}^{\bullet}O_2^{-}$-scavenging assay

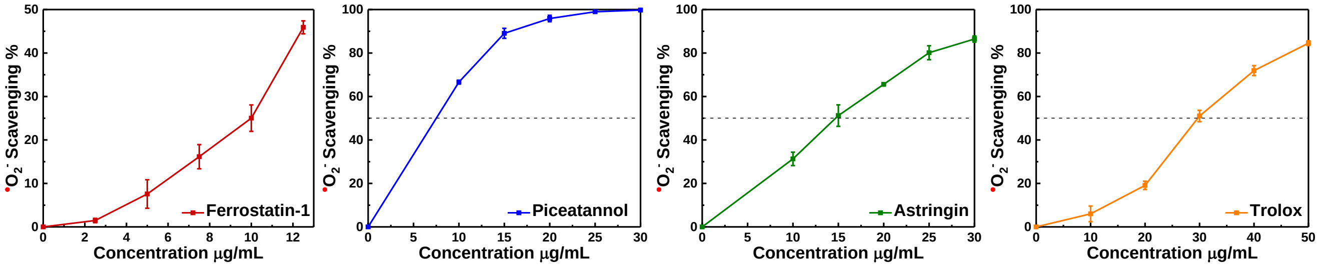

Fig. S1.1 The does response curves of Ferrostatin-1, piceatannol, astringin, and Trolox in ${}^{\bullet}O_2^{-}$-scavenging assay.

Tab. S1.1 The comparison of IC_{50} values of Ferrostatin-1, piceatannol, astringin, and Trolox in ${}^{\bullet}O_2^{-}$-scavenging assay.

	Ferrostatin-1	Piceatannol	Astringin	Trolox
μg/mL	15.3±0.7	7.5±0.1	15.4±0.1	31.8±1.1
μmol/L	58.2±2.7 ^{b}	30.7±0.4 ^{a}	37.8±0.3 ^{a}	127.0±4.2 ^{c}

The IC_{50} value was defined as the final concentration of 50% scavenging power, calculated by linear regression analysis, and expressed as the mean ± SD (n = 3). The linear regression was analyzed by the Origin 2017 professional software. The IC_{50} values with different superscripts (^{a}, ^{b}, or ^{c}) in the same row, are significantly different ($p < 0.05$). Trolox is the positive control.

2. *PTIO•-scavenging assay (pH 4.5)*

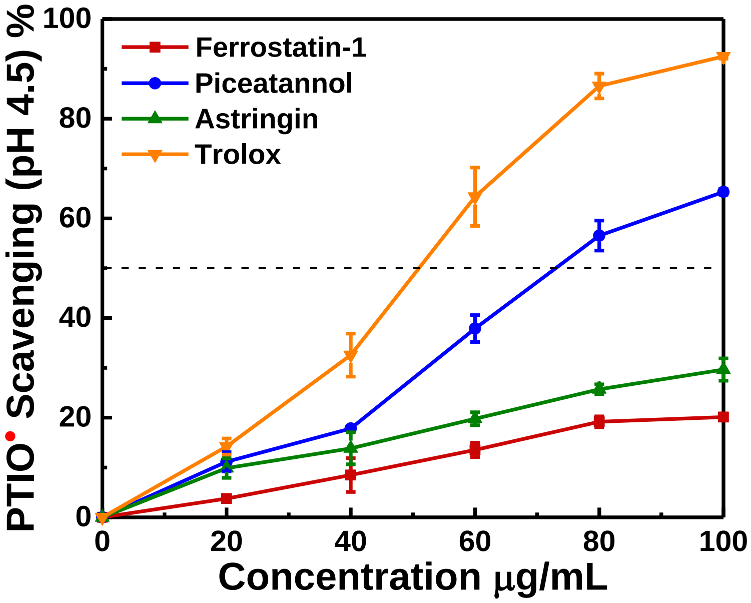

Fig. S1.2 The does response curves of Ferrostatin-1, piceatannol, astringin, and Trolox in PTIO•-scavenging (pH 4.5) assay.

Tab. S1.2 The comparison of IC_{50} values of Ferrostatin-1, piceatannol, astringin, and Trolox in PTIO•-scavenging (pH 4.5) assay.

	Ferrostatin-1	Piceatannol	Astringin	Trolox
μg/mL	230.6±8.7	76.3±2.9	167.1±10.4	51.6±2.1
μmol/L	878.9±33.0 [d]	312.5±12.0 [b]	411.1±25.6 [c]	206.2±8.5 [a]

The IC_{50} value was defined as the final concentration of 50% scavenging power, calculated by linear regression analysis, and expressed as the mean ± SD (n = 3). The linear regression was analyzed by the Origin 2017 professional software. The IC_{50} values with different superscripts ([a], [b], [c], or [d]) in the same row, are significantly different ($p < 0.05$). Trolox is the positive control.

3. *PTIO•-scavenging assay (pH 7.4)*

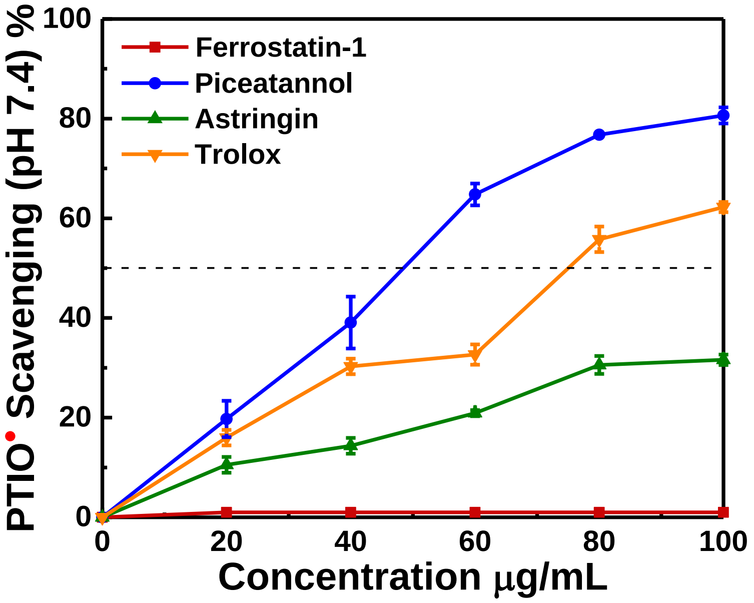

Fig. S1.2 The does response curves of Ferrostatin-1, piceatannol, astringin, and Trolox in PTIO•-scavenging (pH 7.4) assay.

Tab. S1.2 The comparison of IC_{50} values of Ferrostatin-1, piceatannol, astringin, and Trolox in PTIO•-scavenging (pH 7.4) assay.

	Ferrostatin-1	Piceatannol	Astringin	Trolox
μg/mL	>10000	53.7±1.7	149.9±5.3	77.8±1.4
μmol/L	>10000	219.8±7.0 [a]	368.8±12.9 [b]	310.7±5.8 [b]

The IC_{50} value was defined as the final concentration of 50% scavenging power, calculated by linear regression analysis, and expressed as the mean ± SD (n = 3). The linear regression was analyzed by the Origin 2017 professional software. The IC_{50} values with different superscripts ([a] or [b]) in the same row, are significantly different ($p < 0.05$). Trolox is the positive control.

4. *Fe^{3+}-reducing assay*

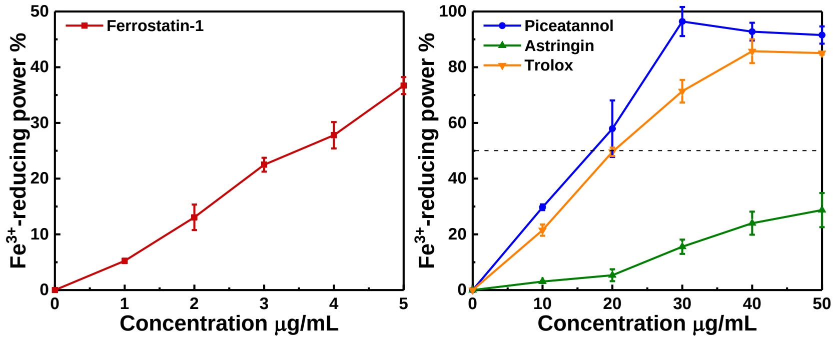

Fig. S1.2 The does response curves of Ferrostatin-1, piceatannol, astringin, and Trolox in Fe^{3+}-reducing assay.

Tab. S1.2 The comparison of IC_{50} values of Ferrostatin-1, piceatannol, astringin, and Trolox in Fe^{3+}-reducing assay.

	Ferrostatin-1	Piceatannol	Astringin	Trolox
μg/mL	6.7±0.1	16.7±1.3	72.7±1.1	22.2±0.4
μmol/L	25.6±0.3 [a]	68.2±5.3 [b]	178.9±2.8 [c]	88.8±1.8 [b]

The IC_{50} value was defined as the final concentration of 50% reducing power, calculated by linear regression analysis, and expressed as the mean ± SD (n = 3). The linear regression was analyzed by the Origin 2017 professional software. The IC_{50} values with different superscripts ([a], [b], or [c]) in the same row, are significantly different ($p < 0.05$). Trolox is the positive control.
