# Supplementary material for: Comparison of Ferroptosis-Inhibitory Mechanisms between Ferrostatin-1 and Dietary Stilbenes (Piceatannol and Astringin)
Source: Molecules. 2021 Feb 19;26(4):1092. doi: 10.3390/molecules26041092 (PMC7922211; doi:10.3390/molecules26041092)
Supplement: Supplementary file 1 [file molecules-26-01092-s001.zip › Suppls/Suppl. 1 Certificates of analysis of stilbenes.pdf]

Supplementary Material-1 Certificates of analysis of stilbenes

Comparison of Ferroptosis-inhibitory Mechanisms between Ferrostatin-1 and Dietary Stilbenes (Piceatannol and Astringin)

Ban Chen [1], Xican Li [1,*], Xiaojian Ouyang [1], Jie Liu [2,3], Yangping Liu [4] Dongfeng Chen[2,*]

1 School of Chinese Herbal Medicine; Guangzhou University of Chinese Medicine, Guangzhou 510006, China; imchenban@foxmail.com (B.C.); oyxiaojian55@163.com (X.O.)

2 School of Basic Medical Science, Guangzhou University of Chinese Medicine, Guangzhou 510006, China; 15014173165@163.com (J.L.)

3. The Research Center of Basic Integrative Medicine, Guangzhou University of Chinese Medicine, Guangzhou 510006, China.

4. The Fourth Clinical Medical College, Guangzhou University of Chinese Medicine, Waihuan East Road No. 232, Guangzhou Higher Education Mega Center, Guangzhou 510006, China; 20182119168@stu.gzucm.edu.cn (Y.L.)

* Correspondence: lixc@gzucm.edu.cn (X.L.); lixican@126.com (X.L.); chen888@gzucm.edu.cn (D.C.). Tel: +86-020-39358076; Fax: +86-020-38892690

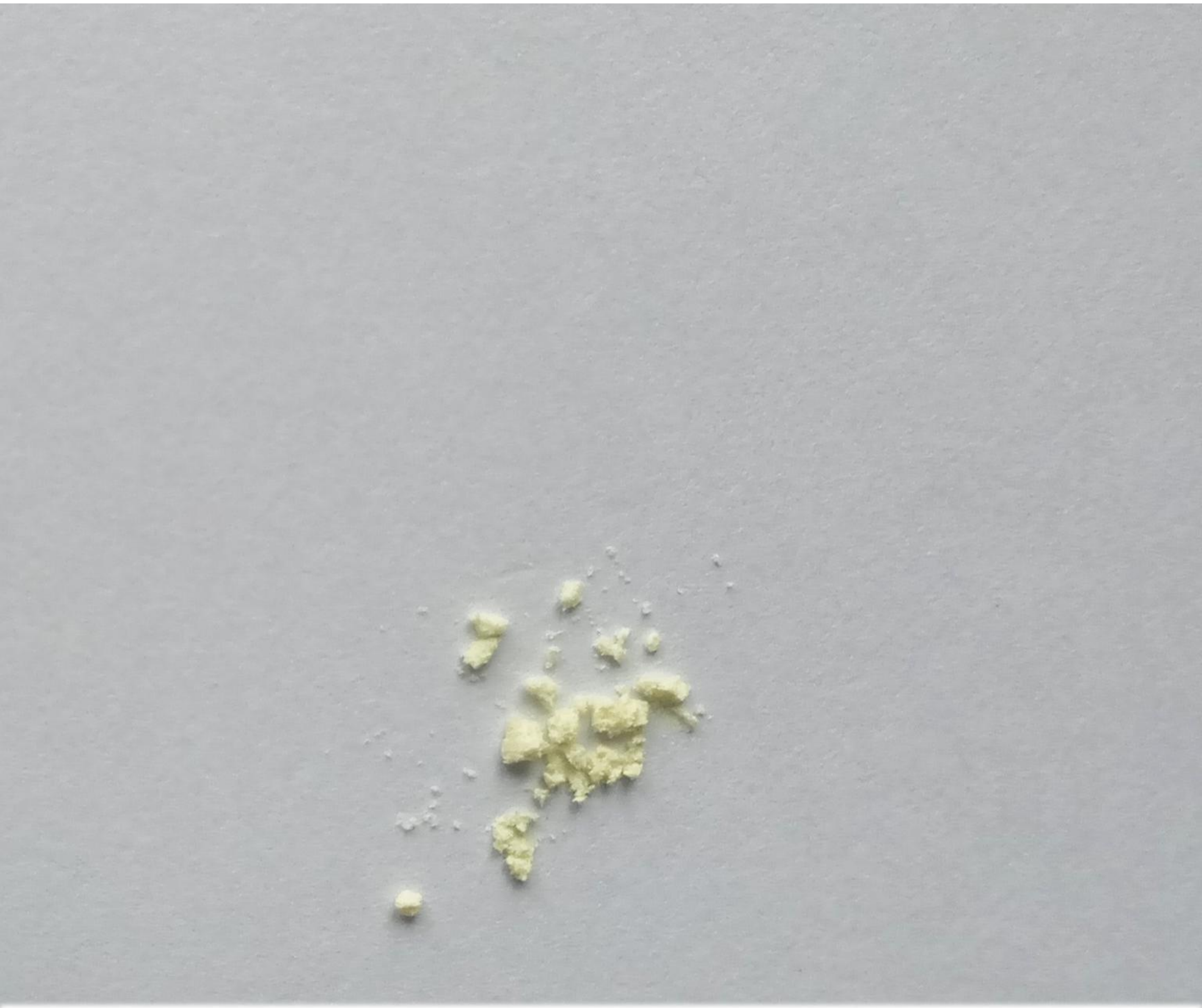

Piceatannol

CAS No.: 10083-24-6

产品分析证书
Certificate of Analysis

中文名称：白皮杉醇

English Name：Piceatannol
别名(Alias):

产品编码(Cat. No.):BP1525
CAS Number: 10083-24-6
分子式(M. F.): C14H12O4
分子量(M. W.): 244.246

批号(Batch No.): PRF9091121
报告日期(Report date): 2018-09-11

Structure

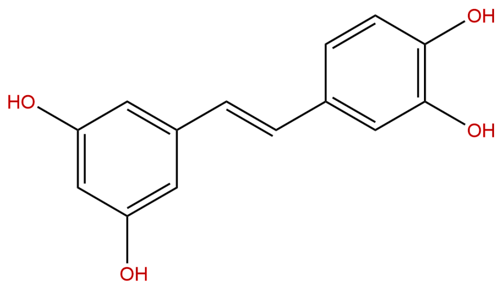

检验结果（Analytical result）:

检验项目（Test Item）	检验指标（Specifications）	检验结果（Results）
外观Appearance	Light yellow powder	Light yellow powder
干燥失重Loss on drying	＜3.0%	1.45%
纯度Purity (HPLC-DAD,325nm)*	≥98.0%	99.06%
质谱Mass	244.2±1	Conforms
核磁NMR	Comply with the structure	Conforms

*** 色谱图见附件（Please find HPLC chromatography attached.）**

检测方法（Test Method）：Column: Thermo HyPURITY C18,4.6*150mm,3.0um; **Column temperature**: 30℃; **Detection Mode:** UV325nm;**Flow Rate:** 1.0ml/min;**Sample dissolution:** Methanol; **Mobile Phase: A,** Acetonitrile **B,** 0.1%Phosphoric acid in water;**Gradient elution:** A, 10%-25%,15min.

贮存条件（Storage）:2~8℃, protected from light, keep package airproofed when not in use.

复测期（Retest date）:Three years (2021-09-10) under conditions list above.

QC: Zhang Ling
Date: 2018-09-11

QA: Meng Pan
Date: 2018-09-11

备注(Remarks):The sample solutions should be prepared and used on the same day, it is the best preparing the solutions immediately before use. If the solutons have to be made up in advance, it should be made as aliquots in tightly sealed vials at less than -20℃. Generally, these might be useable for up to two weeks. The quality of the product is only guaranteed to comply with the attached COA.
In case of quality issue, please contact us within 15 days after receipt of the product.

Tel：+86-28-82633397　　Fax：+86-28-82633165
http://www.phytopurify.com　　Email：sales@biopurify.com　　biopurify@gmail.com

Chengdu Biopurify Phytochemicals Ltd HPLC Report

Injection Details

Injection Name:	**Piceatannol PRF9091121**	*Run Time (min):*	**25.00**
Vial Number:	**GA5**	*Injection Volume:*	**10.00**
Injection Type:	**Unknown**	*Channel:*	**UV_VIS_1**
Calibration Level:		*Wavelength:*	**325**
Instrument Method:	**Piceatannol**	*Bandwidth:*	**4**
Processing Method:	**Piceatannol**	*Dilution Factor:*	**1.0000**
Injection Date/Time:	**11/09/2018 13:44**	*Sample Weight:*	**1.0000**

Chromatogram

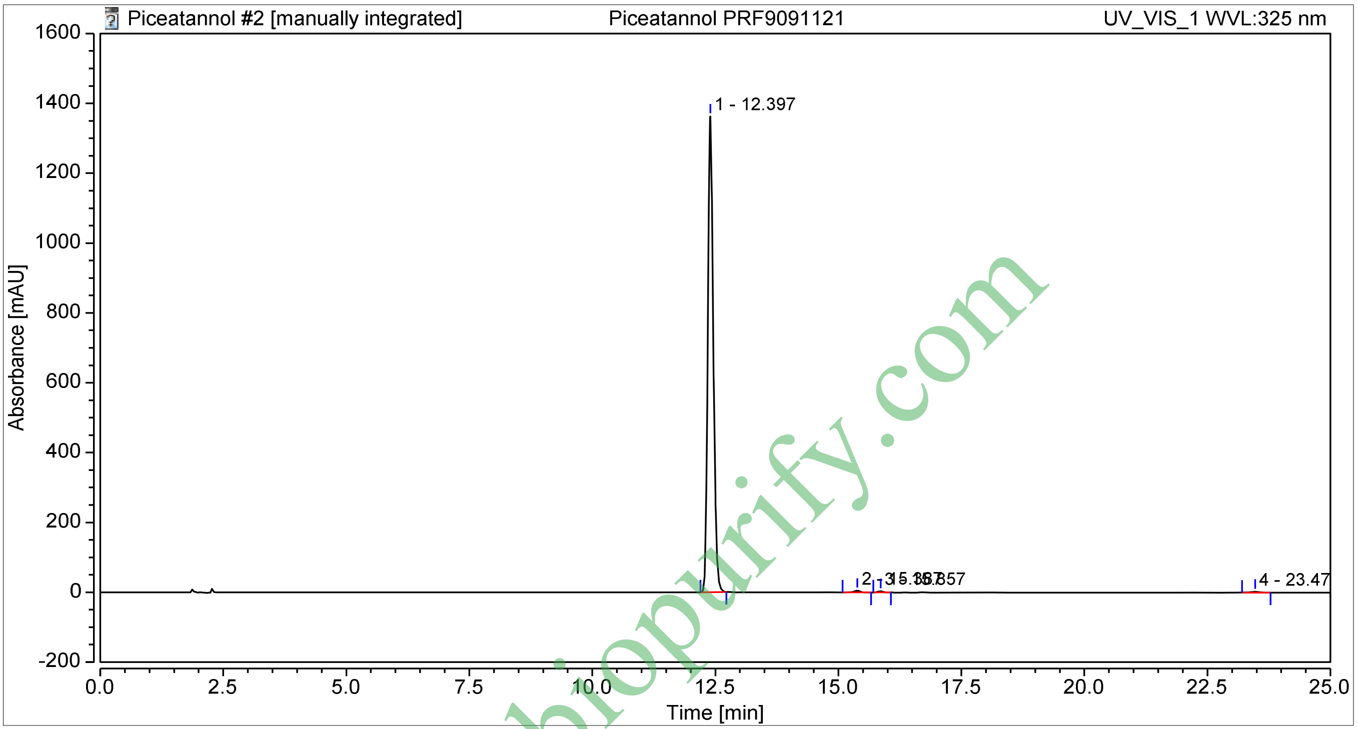

www.biopurify.com

Integration Results

No.	Retention Time	Area	Height	Relative Area	Plates (USP)	Resolution (USP)	Asymmetry
	min	mAU*min	mAU	%			
1	12.397	177.054	1362.501	99.06	59329	14.44	1.19
2	15.387	0.728	5.211	0.41	85363	2.33	1.08
3	15.857	0.455	3.649	0.25	108533	27.81	1.26
4	23.470	0.506	2.191	0.28	69964	n.a.	1.05
**Total:**		**178.743**	**1373.553**	**100.00**			**0.00**

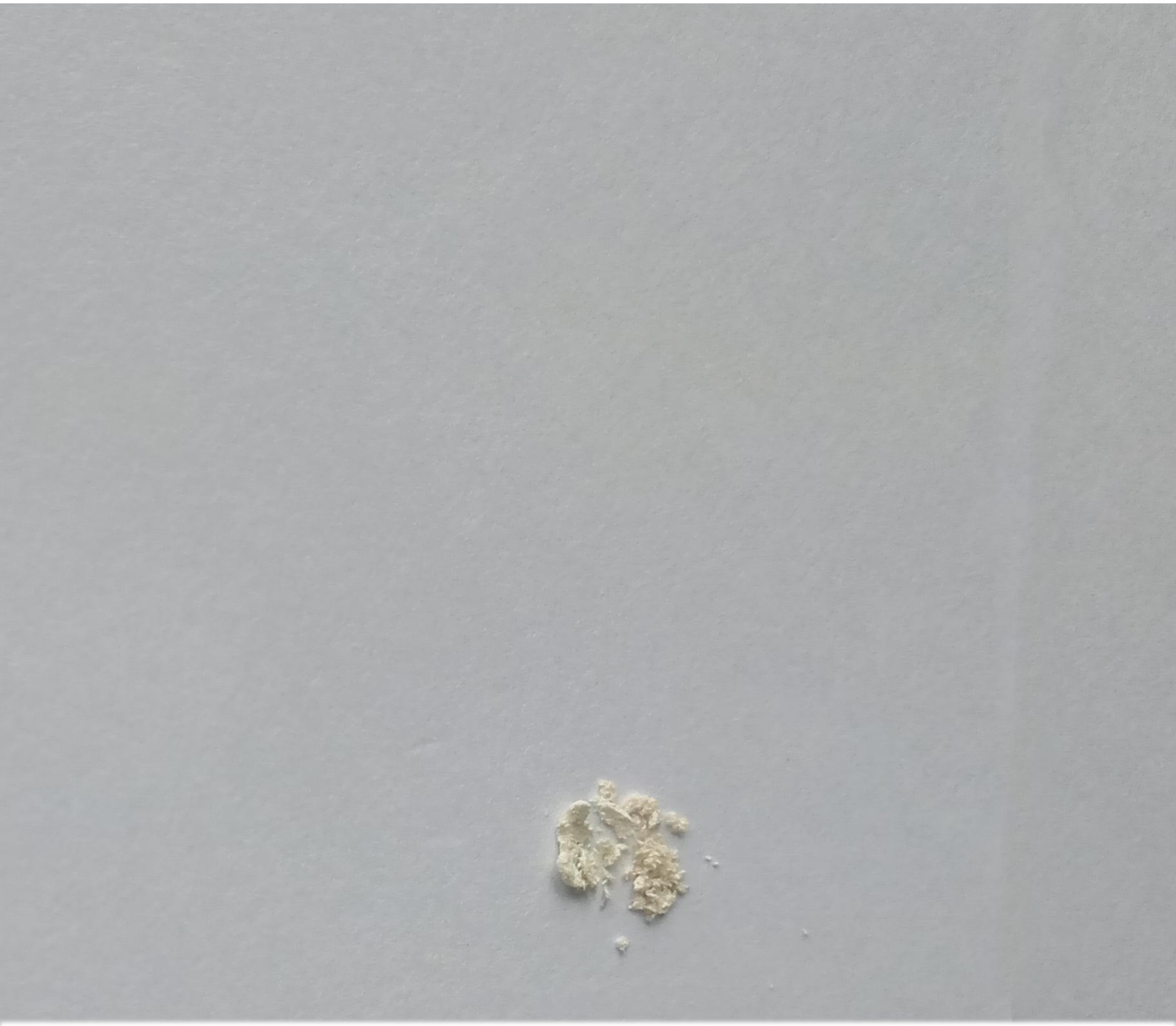

Astringin

CAS No.: 29884-49-9

ChemFaces®

ISO 9001:2015 Certification
www.chemfaces.com

CERTIFICATE OF ANALYSIS

Astringin

[**Catalog No.**] CFN92486

[**CAS No.**] 29884-49-9

[**Purity**] >=98%

[**M.F**] $C_{20}H_{22}O_9$

[**Physical Description**] Powder

[**Solvent**] DMSO, Pyridine, Methanol, Ethanol, etc.

[**Weight**] 10mg

[**Lot No.**] CFS201901

[**Storage**] Protected from air and light, refrigerate or freeze(2-8 °C)

[**Intended Use**] For laboratory use only

[**Shelf Life**] 2 years

[**Chemical Structure**]

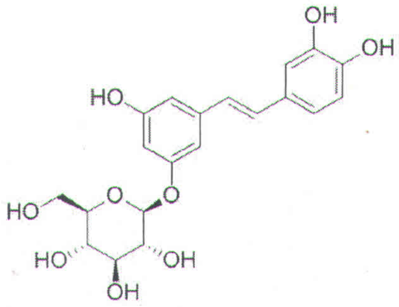

[**Characterization Data Summary**]

Analytical Test	Results
Identification by ^{1}H-NMR, HPLC	Consistent with the above structure
Purity tested	>=98%

Authorized Signature:

Wuhan ChemFaces Biochemical Co., Ltd.
Q. C.
PASS

Date: Oct 09, 2020

www. chemfaces. com
No.176 Checheng East Rd., Wuhan Economic and Technological Development Zone, Wuhan, Hubei 430056, PRC
Email: manager@chemfaces.com | Tech: service@chemfaces.com | Tel: 0086-27-84237683

Analysis Report

<Sample Information>

Sample Name : CFN92486
Instrument : SHIMADZU LC-20AT
Column : Wonda Cract ODS-2 (5 μm, 4.6×250 mm)
Column Temp. : 35°C
Mobile phase flow : 1.0 ml/min
Injection Volume : 5 uL
Detection Wave. : UV-276nm
Con. & Solvent : 1mg/ml (Methanol)

<Analysis condition>

5%→100% Acetonitrile in 0.05% Phosphoric acid H_2O
over 10.0min followed by 100% Acetonitrile to 17.0 min

<Chromatogram>

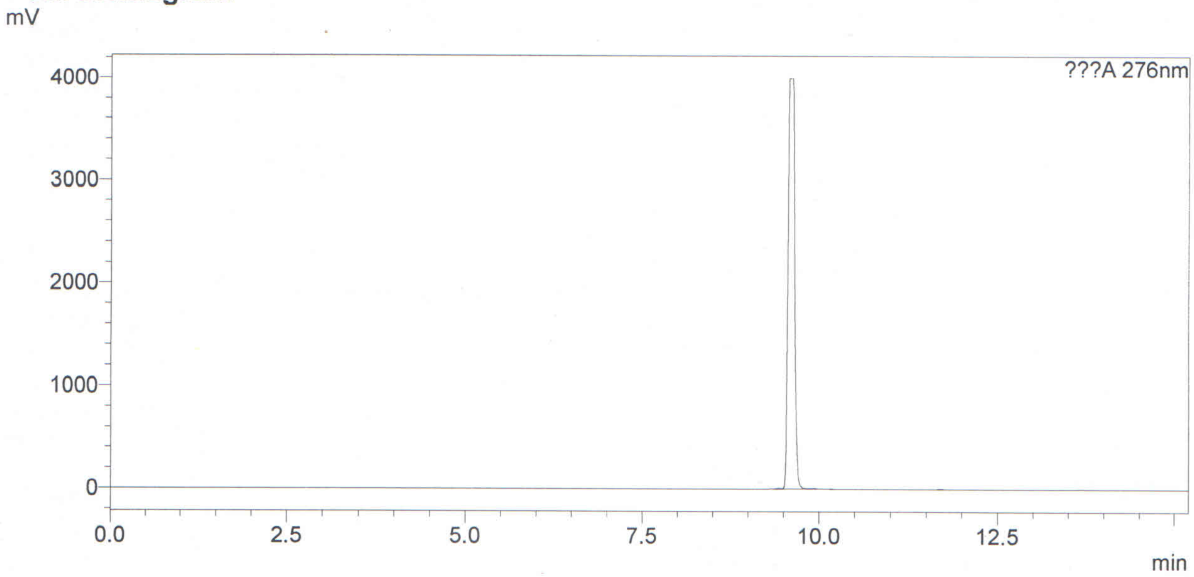

<Peak Table>

???A 276nm

Peak#	Ret. Time	Area	Height	Conc.	Unit	Area%
1	9.442	39689	7201	0.155		0.155
2	9.579	25478593	3998904	99.649		99.649
3	9.924	28244	5818	0.110		0.110
4	11.701	21818	4614	0.085		0.085
Total		25568344	4016538			100.000

www. chemfaces. com
No.176 Checheng East Rd., Wuhan Economic and Technological Development Zone, Wuhan, Hubei 430056, PRC
Email: manager@chemfaces.com | Tech: service@chemfaces.com | Tel: 0086-27-84237683

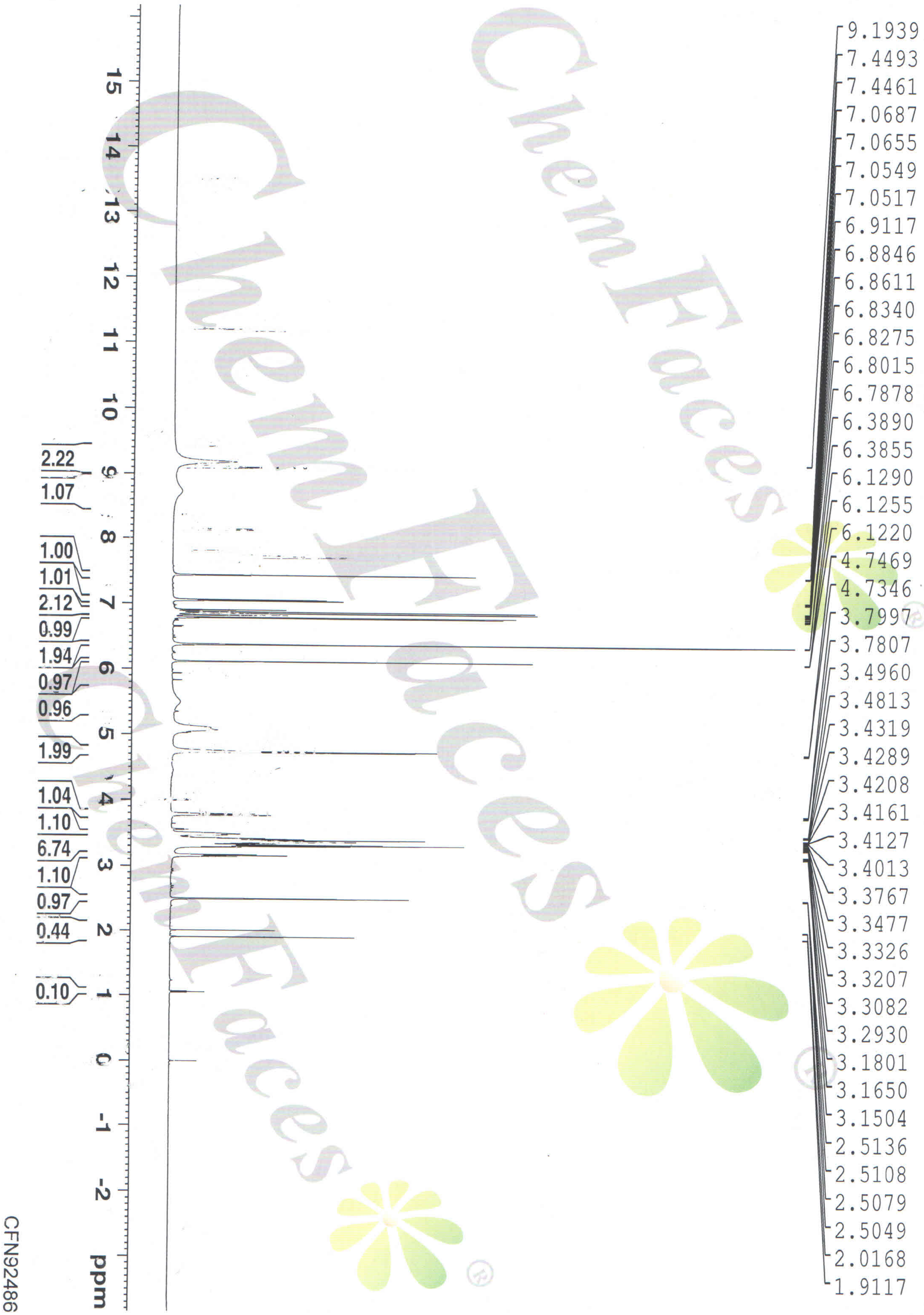
9.1939
7.4493
7.4461
7.0687
7.0655
7.0549
7.0517
6.9117
6.8846
6.8611
6.8340
6.8275
6.8015
6.7878
6.3890
6.3855
6.1290
6.1255
6.1220
4.7469
4.7346
3.7997
3.7807
3.4960
3.4813
3.4319
3.4289
3.4208
3.4161
3.4127
3.4013
3.3767
3.3477
3.3326
3.3207
3.3082
3.2930
3.1801
3.1650
3.1504
2.5136
2.5108
2.5079
2.5049
2.0168
1.9117
2.22
1.07
1.00
1.01
2.12
0.99
1.94
0.97
0.96
1.99
1.04
1.10
6.74
1.10
0.97
0.44
0.10
ppm
CFN92486
