# Supplementary material for: Comparison of Ferroptosis-Inhibitory Mechanisms between Ferrostatin-1 and Dietary Stilbenes (Piceatannol and Astringin)
Source: Molecules. 2021 Feb 19;26(4):1092. doi: 10.3390/molecules26041092 (PMC7922211; doi:10.3390/molecules26041092)
Supplement: Supplementary file 1 [file molecules-26-01092-s001.zip › Suppls/Suppl. 3 The partial calculation results of neutral molecules.pdf]

Supplementary Material-3 The partial calculation results of neutral molecules

Comparison of Ferroptosis-inhibitory Mechanisms between Ferrostatin-1 and Dietary Stilbenes (Piceatannol and Astringin)

Ban Chen [1], Xican Li [1,*], Xiaojian Ouyang [1], Jie Liu [2,3], Yangping Liu [4] Dongfeng Chen[2,*]

1 School of Chinese Herbal Medicine; Guangzhou University of Chinese Medicine, Guangzhou 510006, China; imchenban@foxmail.com (B.C.); oyxiaojian55@163.com (X.O.)

2 School of Basic Medical Science, Guangzhou University of Chinese Medicine, Guangzhou 510006, China; 15014173165@163.com (J.L.)

3. The Research Center of Basic Integrative Medicine, Guangzhou University of Chinese Medicine, Guangzhou 510006, China.

4. The Fourth Clinical Medical College, Guangzhou University of Chinese Medicine, Waihuan East Road No. 232, Guangzhou Higher Education Mega Center, Guangzhou 510006, China; 20182119168@stu.gzucm.edu.cn (Y.L.)

* Correspondence: lixc@gzucm.edu.cn (X.L.); lixican@126.com (X.L.); chen888@gzucm.edu.cn (D.C.). Tel: +86-020-39358076; Fax: +86-020-38892690

Table S3.1 The partial calculation results of Ferrostatin-1

Ferrostatin-1 calculated at M06-2X-D3/6-311+G** level in methanol SMD solvation model					
Cartesian coordinates of optimized molecule				Thermal properties /(Hartree)	
Symbol	X	Y	Z	Zero-point correction	0.358188
C	3.785627	-1.237323	1.669815	Thermal correction to Energy	0.376542
C	4.936856	-0.234628	1.575344	Thermal correction to Enthalpy	0.377486
C	4.171802	0.411268	-0.736583	Thermal correction to Gibbs Free Energy	0.310586
C	3.031282	-0.604455	-0.643158	Sum of electronic and zero-point Energies	-844.367961
C	2.594199	-0.806794	0.808483	Sum of electronic and thermal Energies	-844.349607
C	-0.391702	-1.044722	-1.271547	Sum of electronic and thermal Enthalpies	-844.348663
C	0.604198	-0.085292	-1.081477	Sum of electronic and thermal Free Energies	-844.415563
N	1.910929	-0.277445	-1.582571	Single point energy at def2-TZVPD basis set	-844.8137027
C	-1.691085	-0.848413	-0.822135		
C	-2.003574	0.335094	-0.153642		
C	-1.02705	1.316065	0.025782		
C	0.270197	1.132308	-0.445253		
C	-3.365652	0.602529	0.370131		
O	-4.232165	-0.373092	0.104676		
C	-5.583443	-0.194543	0.585071		
C	-6.366582	-1.417315	0.175224		
N	1.243773	2.113244	-0.275534		
O	-3.676628	1.605347	0.982019		
C	5.361502	-0.029184	0.120302		
H	3.464464	-1.352597	2.708825		
H	4.136705	-2.220544	1.333035		
H	4.612541	0.726269	1.993948		
H	5.785324	-0.575913	2.175477		
H	4.469721	0.523807	-1.783289		
H	3.823604	1.388022	-0.384424		
H	3.432154	-1.56436	-0.991002		
H	2.18307	0.129511	1.201763		
H	1.80271	-1.560622	0.857465		
H	-0.127037	-1.968636	-1.776122		
H	1.878667	-0.997085	-2.29499		
H	-2.442002	-1.612582	-0.975025		
H	-1.284829	2.248585	0.516768		
H	-5.550579	-0.074981	1.669576		
H	-5.988786	0.717162	0.142378		
H	-7.397076	-1.318486	0.522305		
H	-6.376635	-1.525405	-0.911227		
H	-5.937778	-2.318633	0.617871		
H	0.864813	3.043913	-0.148174		
H	1.952061	2.094524	-1.001507		

H	6.162719	0.712274	0.055721		
H	5.764159	-0.970444	-0.274323		
Ferrostatin-1 calculated at M06-2X-D3/6-311+G** level in aqueous SMD solvation model					
Cartesian coordinates of optimized molecule				Thermal properties /(Hartree)	
Symbol	X	Y	Z	Zero-point correction	0.358268
C	3.783834	-1.281385	1.639738	Thermal correction to Energy	0.376623
C	4.916914	-0.255817	1.584271	Thermal correction to Enthalpy	0.377568
C	4.158778	0.447719	-0.712533	Thermal correction to Gibbs Free Energy	0.310677
C	3.036447	-0.590073	-0.658742	Sum of electronic and zero-point Energies	-844.358591
C	2.592474	-0.845487	0.78185	Sum of electronic and thermal Energies	-844.340236
C	-0.386629	-1.033411	-1.280897	Sum of electronic and thermal Enthalpies	-844.339291
C	0.609869	-0.075245	-1.08519	Sum of electronic and thermal Free Energies	-844.406182
N	1.912581	-0.269752	-1.598899	Single point energy at def2-TZVPD basis set	-844.8044063
C	-1.685366	-0.842787	-0.82882		
C	-1.99864	0.335326	-0.151104		
C	-1.02122	1.313564	0.035698		
C	0.276216	1.134727	-0.436189		
C	-3.360015	0.599278	0.374488		
O	-4.228292	-0.374992	0.102925		
C	-5.579341	-0.195623	0.585313		
C	-6.367981	-1.411988	0.167812		
N	1.244406	2.119614	-0.251327		
O	-3.671026	1.59899	0.993488		
C	5.348663	-0.002464	0.139201		
H	3.457228	-1.434575	2.671639		
H	4.155197	-2.246307	1.274252		
H	4.570854	0.686772	2.025637		
H	5.76627	-0.598159	2.18166		
H	4.464146	0.597371	-1.751789		
H	3.79665	1.407387	-0.330817		
H	3.460891	-1.529252	-1.031534		
H	2.161035	0.070554	1.20032		
H	1.813919	-1.613677	0.799344		
H	-0.12187	-1.951995	-1.79439		
H	1.866833	-1.016132	-2.282432		
H	-2.434867	-1.6065	-0.987798		
H	-1.276884	2.242309	0.534299		
H	-5.545017	-0.084182	1.670156		
H	-5.981236	0.720226	0.149308		
H	-7.396991	-1.310133	0.516885		
H	-6.379939	-1.512194	-0.918891		
H	-5.943181	-2.317808	0.603876		
H	0.853771	3.046253	-0.131565		

H	1.96013	2.10728	-0.969247		
H	6.14004	0.750604	0.101619		
H	5.764641	-0.926882	-0.279627		

Table S3.2 The partial calculation results of piceatannol

Piceatannol calculated at M06-2X-D3/6-311+G** level in methanol SMD solvation model					
Cartesian coordinates of optimized molecule				Thermal properties /(Hartree)	
Symbol	X	Y	Z	Zero-point correction	0.232282
C	-0.381757	0.298187	-0.011406	Thermal correction to Energy	0.248535
C	0.627627	-0.583002	-0.040316	Thermal correction to Enthalpy	0.249479
C	-1.815117	-0.025904	-0.025349	Thermal correction to Gibbs Free Energy	0.187300
C	2.061153	-0.252488	-0.026584	Sum of electronic and zero-point Energies	-841.309835
C	-2.732209	1.026636	0.099827	Sum of electronic and thermal Energies	-841.293582
C	-2.310634	-1.328993	-0.15998	Sum of electronic and thermal Enthalpies	-841.292638
C	2.972718	-1.306907	0.064253	Sum of electronic and thermal Free Energies	-841.354817
C	2.537221	1.065093	-0.100476	Single point energy at def2-TZVPD basis set	-841.6364985
C	-4.097085	0.790216	0.096687		
C	-3.678673	-1.56849	-0.161977		
C	4.341484	-1.04546	0.089607		
C	3.904652	1.300794	-0.073725		
O	-4.959815	1.839642	0.220795		
C	-4.576788	-0.516134	-0.03378		
O	5.178263	-2.118479	0.185051		
C	4.823078	0.254997	0.021049		
O	4.421194	2.561794	-0.140699		
O	-5.933603	-0.659799	-0.027345		
H	-0.161652	1.361875	0.035245		
H	0.411727	-1.647855	-0.064394		
H	-2.384742	2.049676	0.202644		
H	-1.635642	-2.169201	-0.268407		
H	2.627993	-2.33336	0.120159		
H	1.861399	1.908756	-0.184225		
H	-4.064384	-2.577492	-0.266842		
H	-5.870245	1.513532	0.197211		
H	6.098877	-1.827092	0.202151		
H	5.88783	0.461547	0.038668		
H	3.707532	3.209872	-0.200587		
H	-6.181013	-1.586787	-0.141013		
Piceatannol calculated at M06-2X-D3/6-311+G** level in aqueous SMD solvation model					
Cartesian coordinates of optimized molecule				Thermal properties /(Hartree)	
Symbol	X	Y	Z	Zero-point correction	0.232129
C	-0.380876	0.296798	-0.010642	Thermal correction to Energy	0.248362
C	0.627485	-0.585484	-0.039011	Thermal correction to Enthalpy	0.249307
C	-1.814084	-0.026984	-0.023253	Thermal correction to Gibbs Free Energy	0.187262
C	2.060592	-0.254187	-0.026168	Sum of electronic and zero-point Energies	-841.304918
C	-2.730256	1.026992	0.094174	Sum of electronic and thermal Energies	-841.288685
C	-2.309498	-1.331051	-0.149015	Sum of electronic and thermal Enthalpies	-841.287741

C	2.973325	-1.308261	0.059437	Sum of electronic and thermal Free Energies	-841.349785
C	2.535023	1.063954	-0.095387	Single point energy at def2-TZVPD basis set	-841.6312705
C	-4.094789	0.790151	0.091169		
C	-3.677415	-1.570081	-0.15127		
C	4.340876	-1.044126	0.083907		
C	3.901967	1.300297	-0.06972		
O	-4.962161	1.844566	0.208286		
C	-4.574603	-0.516027	-0.031606		
O	5.185451	-2.119253	0.175194		
C	4.822274	0.256435	0.019269		
O	4.415551	2.569345	-0.13245		
O	-5.936024	-0.662816	-0.02673		
H	-0.160511	1.360356	0.033558		
H	0.41162	-1.650185	-0.062492		
H	-2.381058	2.04972	0.190087		
H	-1.634037	-2.171528	-0.249572		
H	2.628478	-2.334484	0.111925		
H	1.858601	1.907467	-0.173221		
H	-4.064994	-2.578687	-0.249255		
H	-5.869545	1.510932	0.184953		
H	6.102119	-1.816302	0.193822		
H	5.886968	0.461549	0.036552		
H	3.693282	3.208405	-0.183922		
H	-6.173576	-1.592524	-0.138142		

Table S3.3 The partial calculation results of astringin

Astringin calculated at M06-2X-D3/6-311+G** level in methanol SMD solvation model					
Cartesian coordinates of optimized molecule				Thermal properties /(Hartree)	
Symbol	X	Y	Z	Zero-point correction	0.408893
C	-3.854124	0.327293	-0.880816	Thermal correction to Energy	0.435952
O	-2.535514	-0.149322	-1.083274	Thermal correction to Enthalpy	0.436897
O	-4.075947	0.741908	0.441277	Thermal correction to Gibbs Free Energy	0.351563
C	-4.801207	-0.788605	-1.326123	Sum of electronic and zero-point Energies	-1451.878359
C	-1.480982	0.68748	-0.835879	Sum of electronic and thermal Energies	-1451.851299
C	-4.127306	-0.26453	1.467166	Sum of electronic and thermal Enthalpies	-1451.850355
C	-4.886142	-1.931439	-0.301145	Sum of electronic and thermal Free Energies	-1451.935689
O	-6.083557	-0.239646	-1.57181	Single point energy at def2-TZVPD basis set	-1452.4415739
C	-0.226275	0.081873	-0.837587		
C	-1.619964	2.050287	-0.587382		
C	-5.107427	-1.380605	1.107001		
C	-2.738354	-0.736831	1.886742		
O	-3.704367	-2.708902	-0.258355		
C	0.920489	0.839741	-0.588804		
C	-0.462539	2.79761	-0.349914		
O	-6.442818	-0.880155	1.10887		
O	-1.977984	0.349687	2.403884		
C	2.218644	0.14795	-0.589802		
C	0.795468	2.213975	-0.347407		
O	-0.528918	4.138222	-0.110189		
C	3.381723	0.670111	-0.177111		
C	4.680668	-0.01733	-0.179802		
C	5.745862	0.57036	0.515844		
C	4.905335	-1.227911	-0.846576		
C	6.989879	-0.037229	0.562693		
C	6.15252	-1.83801	-0.802735		
C	7.196671	-1.251712	-0.098315		
O	8.001267	0.561597	1.254755		
O	8.45104	-1.780393	-0.004927		
H	-4.030914	1.212605	-1.496062		
H	-4.433762	-1.182044	-2.276475		
H	-4.543294	0.270458	2.326243		
H	-5.749983	-2.550499	-0.565297		
H	-6.516165	-0.090029	-0.717417		
H	-0.157613	-0.983295	-1.028627		
H	-2.58476	2.541361	-0.567248		
H	-5.002249	-2.196048	1.829242		
H	-2.203538	-1.2283	1.073355		
H	-2.855061	-1.450986	2.704502		

H	-3.548929	-3.094627	-1.129028		
H	-6.637641	-0.490501	1.969899		
H	-1.81666	0.980945	1.69017		
H	2.18501	-0.879843	-0.941633		
H	1.658069	2.843122	-0.164855		
H	-1.445823	4.44072	-0.12799		
H	3.403658	1.681862	0.220601		
H	5.609758	1.512671	1.036884		
H	4.113179	-1.699074	-1.415804		
H	6.330687	-2.774293	-1.322023		
H	8.798004	0.015993	1.200926		
H	8.504774	-2.619047	-0.481483		
Astringin calculated at M06-2X-D3/6-311+G** level in aqueous SMD solvation model					
Cartesian coordinates of optimized molecule				Thermal properties /(Hartree)	
Symbol	X	Y	Z	Zero-point correction	0.408866
C	-3.863986	0.334769	-0.88236	Thermal correctio n to Energy	0.436084
O	-2.54708	-0.137667	-1.108184	Thermal correction to Enthalpy	0.437028
O	-4.067761	0.742347	0.446787	Thermal correction to Gibbs Free Energy	0.351168
C	-4.81406	-0.782134	-1.317611	Sum of electronic and zero-point Energies	-1451.873802
C	-1.49012	0.695675	-0.848642	Sum of electronic and thermal Energies	-1451.846584
C	-4.09707	-0.270494	1.467243	Sum of electronic and thermal Enthalpies	-1451.845640
C	-4.878324	-1.931238	-0.298134	Sum of electronic and thermal Free Energies	-1451.931500
O	-6.103863	-0.236836	-1.536312	Single point energy at def2-TZVPD basis set	-1452.4367737
C	-0.237565	0.086071	-0.841347		
C	-1.627555	2.057744	-0.596997		
C	-5.07543	-1.391105	1.117633		
C	-2.70001	-0.740274	1.861727		
O	-3.696961	-2.712027	-0.286326		
C	0.909086	0.839578	-0.579714		
C	-0.470172	2.798944	-0.343504		
O	-6.415659	-0.901894	1.149479		
O	-1.929785	0.34687	2.365088		
C	2.205414	0.145066	-0.577739		
C	0.785903	2.212715	-0.330803		
O	-0.537864	4.144541	-0.097368		
C	3.369179	0.666979	-0.166839		
C	4.667379	-0.021209	-0.171805		
C	5.737693	0.574932	0.508303		
C	4.886545	-1.239119	-0.827197		
C	6.981984	-0.031739	0.549869		
C	6.134539	-1.847457	-0.788705		
C	7.184106	-1.251904	-0.100278		
O	8.004549	0.576614	1.229361		

O	8.443727	-1.781959	-0.014328		
H	-4.05168	1.223359	-1.488651		
H	-4.461641	-1.170025	-2.275554		
H	-4.502027	0.256103	2.336337		
H	-5.74663	-2.547902	-0.551169		
H	-6.518256	-0.102221	-0.670671		
H	-0.169051	-0.978185	-1.035754		
H	-2.590269	2.552742	-0.587883		
H	-4.949358	-2.20932	1.832795		
H	-2.179688	-1.230335	1.038392		
H	-2.802837	-1.455277	2.679946		
H	-3.547513	-3.060111	-1.173584		
H	-6.582952	-0.495181	2.008158		
H	-1.777984	0.971219	1.643219		
H	2.170157	-0.882457	-0.929563		
H	1.648933	2.837083	-0.136086		
H	-1.456209	4.440983	-0.128491		

Table S3.4 The partial calculation results of cytosine, adenine, and guanine

Cytosine calculated at M06-2X-D3/6-311+G** level in aqueous SMD solvation model					
Cartesian coordinates of optimized molecule				Thermal properties /(Hartree)	
Symbol	X	Y	Z	Zero-point correction	0.098972
N	-1.277274	0.885239	-0.000408	Thermal correction to Energy	0.105594
C	-1.172252	-0.497976	0.000001	Thermal correction to Enthalpy	0.106539
O	-2.214319	-1.171305	-0.000007	Thermal correction to Gibbs Free Energy	0.068305
N	0.062698	-1.050556	0.00051	Sum of electronic and zero-point Energies	-394.829026
C	1.133801	-0.258962	0.000098	Sum of electronic and thermal Energies	-394.822404
N	2.340002	-0.838161	-0.000073	Sum of electronic and thermal Enthalpies	-394.821460
C	1.04922	1.171107	0.000219	Sum of electronic and thermal Free Energies	-394.859694
C	-0.192625	1.700688	0.000033	Single point energy at def2-TZVPD basis set	-394.9725806
H	-2.211417	1.277331	-0.000567		
H	2.41607	-1.844755	-0.001446		
H	3.179651	-0.279061	-0.001079		
H	1.935566	1.788168	0.000519		
H	-0.392172	2.763958	0.000324		
Adenine calculated at M06-2X-D3/6-311+G** level in aqueous SMD solvation model					
Cartesian coordinates of optimized molecule				Thermal properties /(Hartree)	
Symbol	X	Y	Z	Zero-point correction	0.112865
N	1.954137	-0.472269	0.000004	Thermal correction to Energy	0.120137
C	1.339431	-1.667449	0.001152	Thermal correction to Enthalpy	0.121081
N	0.044234	-1.930295	0.002199	Thermal correction to Gibbs Free Energy	0.081104
C	-0.718262	-0.818152	0.001631	Sum of electronic and zero-point Energies	-467.185678
C	-0.191016	0.469621	-0.000782	Sum of electronic and thermal Energies	-467.178405
C	1.202874	0.634703	-0.002459	Sum of electronic and thermal Enthalpies	-467.177461
N	1.80314	1.843638	-0.043964	Sum of electronic and thermal Free Energies	-467.217438
N	-2.092006	-0.769056	0.002452	Single point energy at def2-TZVPD basis set	-467.3512634
C	-2.372917	0.516885	0.000129		
N	-1.278513	1.312944	-0.001881		
H	2.004962	-2.523583	0.002758		
H	1.26578	2.673972	0.159218		
H	2.79699	1.881003	0.133925		
H	-3.367929	0.93493	-0.000428		
H	-1.277415	2.325288	-0.005171		
Guanine calculated at M06-2X-D3/6-311+G** level in aqueous SMD solvation model					
Cartesian coordinates of optimized molecule				Thermal properties /(Hartree)	
Symbol	X	Y	Z	Zero-point correction	0.117547
N	1.739165	-1.486974	0.001485	Thermal correction to Energy	0.125758
C	2.702896	-0.51109	0.00275	Thermal correction to Enthalpy	0.126702
N	2.20507	0.6958	0.001038	Thermal correction to Gibbs Free Energy	0.084548
C	0.532421	-0.855383	-0.001379	Sum of electronic and zero-point Energies	-542.435089
N	-0.684759	-1.442403	0.001174	Sum of electronic and thermal Energies	-542.426878

C	-1.668495	-0.572417	-0.002349	Sum of electronic and thermal Enthalpies	-542.425934
N	-2.952309	-0.992007	-0.053814	Sum of electronic and thermal Free Energies	-542.468087
N	-1.472226	0.785044	-0.001601	Single point energy at def2-TZVPD basis set	-542.613847
C	-0.233542	1.433536	-0.000249		
O	-0.179404	2.663781	0.00223		
C	0.837584	0.496952	-0.001709		
H	1.897622	-2.487315	0.002382		
H	3.752379	-0.760314	0.005048		
H	-3.110241	-1.96918	0.148595		
H	-3.686952	-0.351589	0.214332		
H	-2.287351	1.392335	-0.008555		
